# US Consumers’ Perceptions of Raw and Cooked Broken Rice

**DOI:** 10.3390/foods10122899

**Published:** 2021-11-23

**Authors:** Matthew G. Richardson, Philip Glen Crandall, Han-Seok Seo, Corliss A. O’Bryan

**Affiliations:** Department of Food Science, University of Arkansas, 2650 North Young Avenue, Fayetteville, AR 72704, USA; mgrich95@gmail.com (M.G.R.); hanseok@uark.edu (H.-S.S.); cobryan@uark.edu (C.A.O.)

**Keywords:** hunger, rice, broken rice, sensory analysis, consumer panel

## Abstract

Rice supplies about 20% of the calories to the world’s consumers. Milling removes the outer husk and bran, breaking about 20% of the rice kernels during the milling process that equates to almost 100,000,000 tons of rice annually. Broken rice is discounted in price by almost half or relegated to non-human consumption. This study seeks to understand why this large percentage of rice production is discounted for human consumption. Consumers who routinely consume rice evaluated raw and cooked rice with 5%, 10%, 20%, 30% and 40% levels of brokens. Sensory analysis indicated the appearance of raw rice with high levels of brokens affected the price consumers were willing to pay. Panelists were not able to discern sensory differences amongst cooked rice samples with different brokens percentages despite an eight-fold difference in brokens (*p* < 0.01). From this, we concluded that the price discounts imposed on broken rice are not because of perceived differences in the eating quality of cooked rice. Overall impression and overall texture were the two most significant determinants in willingness to purchase rice. The five cooked-rice samples with different levels of broken rice inclusion did not differ in terms of willingness to purchase.

## 1. Introduction

Rice provides an average of 20% of the calories for millions of persons living in poverty and is a foundational grain for building global food security [1,2]. About 496 million metric tons of rice were milled in 2020 [3], with China and India accounting for almost half of this rice processing [4]. Milling rice removes the outer hull, the nutrient rich bran layer and germ from patty or rough rice, breaking a significant percentage of the whole kernels in the process [5]. In the United States, broken rice (brokens) is defined as rice kernels that are less than 75% of the length of the whole rice kernels [6]. Because brokens are often perceived as having a “poor” appearance, rice with a high percentage of broken kernels demands a significantly lower price at retail. For example, in 2020, broken rice sold at a discount of almost half (42%) of whole kernel counterparts [7]. Although some brokens are routinely mixed into whole rice at retail, brokens are also used for non-human consumption in pet foods, breweries, skin creams, rice flours (ground brokens), and pastas, breads, and cereals extruded from rice flour [8]. Aside from being ground into flour for human consumption, brokens often go to uses that do not utilize their inherent nutrition. Brokens are equal in nutrition to full-length rice kernels [9].

Rice kernels fracture mainly during the milling process because of internal fissuring typically beginning much earlier during the drying process [10]. Increased brokens can be the result of rapid moisture absorption, rapid drying, chalkiness, underdeveloped kernels, and environmental factors such as relative humidity, sudden temperature changes, insect infestation or excessive milling [11]). Modern rice mills typically generate between 10 to 15% brokens, whereas smaller, generally less-modern mills, typically found in low-income countries, can generate as much as 30% brokens [4]. Using a conservative figure of 20% brokens generated world-wide during milling, this equates to almost 100,000,000 tons annually of fully nutritious rice that is either discounted by almost half or relegated to non-human consumption.

In the United States, rice futures are traded on an average of 70% yield of milled rice (MRY) from intact, patty rice. The rice futures market assumes that 55% of the initial mass is whole kernels or head rice (HRY). The ratio 55/70 (HRY/MRY) is the standard on which the futures contracts are bought and sold in the United Sates. This means that 15% of the mass are expected to be typically broken kernels. This is in sharp contrast to some import specification that allow for no more than 4% broken kernels. Understanding that brokens offer significantly lower returns to the rice grower and processor than whole kernels is a vital economic concern for the worldwide rice industry. (Information gained through personal communication with rice industry executive).

Broken rice can bring unique characteristics to cooked rice. Previous studies have shown that adjusting the ratio of broken kernels can influence the texture of the cooked rice [12,13]. Brokens have a larger surface area due to the endosperm being exposed, which increases the capacity for water absorption, causing decreased hardness [14]. A higher percentage of broken rice has been found to increase the release of flavor volatiles [15]. These unique attributes have caused broken rice to be seen as a delicacy in some cultures where consumers are willing to pay a premium for broken rice [16]. More broken rice also increases the amount of starch leached into solution which creates a more viscous texture in the final cooked rice under the same gelatinization temperature and cooking duration [17].

Rice preferences vary widely among consumers based on flavor, aroma, texture, functionality, and appearance [18,19]. Additionally, social and economic factors such as brand value, price, and country of origin all play a role in consumers’ preferences in rice [20,21,22]. Although some cultures eat rice as a side dish and prepare it simply with water and salt, others want or need to eat rice as a main dish, so they flavor it with other aromatics, vegetables, and sauces [23]. In addition, the cooking methods of rice vary greatly depending on preferences and type of rice that is being cooked [24,25]. Optimal water to rice ratios (W/R) vary greatly ranging from 0.8:1 parts of water to rice to 2.5:1 [18,26]. Because of the generally bland flavor of rice, the texture attributes of hardness to stickiness ratio have been found to be a strong indicator of overall acceptability [19,27], which is not to say that other factors such as flavor, aroma, and appearance do not play an important role [28]. These characteristics can be measured analytically [29,30] and with a combination of sensory and analytical methods [31,32]. Preferences, both personal and cultural, may have an impact on brokens’ perception that does not have to be negative, despite brokens being sold at a reduced cost.

The hypothesis for this study is that it is the appearance of brokens in raw rice that deters consumers’ desirability rather than the brokens’ sensory attributes after cooking. Food choices, not just in rice, are a function of many interdependent environmental and personal reasons [33]. The identification of food products that are nutrient rich and affordable should be prioritized in fighting the social inequalities that cause low-income consumers to purchase less healthy foods [34]. This is especially true for rice that is an established food among much of the world’s poorest consumers.

To the best of our knowledge, no prior studies have been conducted that compared the perception of consumers who eat rice at least twice a month in side-by-side comparisons of varying levels of brokens in both the pre-cooking (raw) and post cooking assessment. While trained panels are useful in rice sensory analysis to determine specific factors and flavors, the focus of this study is what drives purchasing decisions of rice consumers therefore untrained participants were recruited [13]. By building on other sensory and market studies that observed how consumers perceive brokens, the objectives of this study were aimed at understanding consumers’ willingness to purchase (WTP) rice with increased levels of brokens was more a factor of the appearance pre-purchase raw rice or sensory attributes of cooked rice. Additional information on WTP with rice consumers in the Philippines is available in Ara [35].

The objective of this study was to determine at what level of brokens (if any) consumers can distinguish noticeable differences in a variety of quality factors in raw and cooked rice. By testing a wide range of percentage brokens (treatments of 5%, 10%, 20%, 30%, and 40%), this study aimed to determine an inflection point at which consumers could distinguish between a standard 5% broken rice and increasing levels of brokens Furthermore, if the percentage broken in the rice was discernible, does this recognition of broken rice affect the consumers’ WTP? By testing subjects from the USA who routinely consume rice on their perception of the raw and cooked rice, this study sought to determine if the varying the levels of broken rice affected various factors in the rice as well as at what point from pre-purchase to post-cooking is the consumers’ preference were most impacted by brokens. By testing a variety of factors at each level of percentage broken, significant drivers of consumers’ WTP could be estimated.

## 2. Materials and Methods

This study was conducted in accordance with the Declaration of Helsinki for studies on human subjects, and the protocol was approved by the Institutional Review Board of the University of Arkansas (Fayetteville, AR, USA). A written informed consent was obtained from each panelist prior to their participation.

### 2.1. Participants

A total of 100 participants were recruited through the consumer profile database of the University of Arkansas Sensory Science Center (Fayetteville, AR, USA). The number of participants is within the accepted range established for consumer testing [36]. A participant’s screener was designed first and foremost to ensure the safety of the sensory personnel, the participants, and the investigators during the COVID-19 pandemic. Questions pertaining to the health, potential exposure to the virus, and travel of the participants ensured that health risks were minimized. The screener asked questions concerning demographics, education, income, allergies, and health conditions. In addition, the screener ensured that the participants were considered rice-eating consumers, which was determined to be consuming rice at least two times per month. The study consisted of 77 females and 23 males aged from 19 years to 64 years (mean age ± standard deviation (SD) = 39.0 ± 11.8). The participants reported as 6 Asian, 10 Black, 7 Hispanic, 3 Other, and 74 White.

### 2.2. Samples and Preparation

To test participants’ perception of uncooked and cooked rice with varying percentages of broken being the only controlled variable, the investigators used the same stock pool of raw rice, commercially used for export, and five levels of brokens prepared from that same stock of raw rice for each portion of the study. Rice was prepared with levels of 5%, 10%, 20%, 30%, and 40% in both cooked and raw rice presentations. The control was set at 5% brokens as that is the generally accepted minimum a consumer would purchase at retail (information gained through personal communication with rice industry executive). This was set as the lower limit of the percentage of brokens. To minimize the effect of other variables, a Satake Test Rice Grader using an indented cylinder grader was used to separate the broken rice from the stock bath or raw rice [37]. The rice was prepared in rice cookers (RC3314W Black & Decker) in intervals so that each cooked rice sample would be served to the group of participants at a consistent temperature of 70 °C ± 2 °C, roughly five to seven minutes apart. In this way, all five participants per session were presented with each of their rice samples at the same time as one another so that they were all roughly the same temperature/freshness. After the rice finished cooking, it was immediately fluffed with a fork and placed into the sample cups until the internal temperature was uniform.

Optimum cooking duration (OCD) was measured by the Ranghino test for milled rice [38]. In a 250 mL beaker, about 100 mL of distilled water was boiled (98 ± 1 °C) and five grams of rice samples were added. Measurement of cooking duration was started immediately. After 10 min and every minute thereafter, 10 individual rice grains were removed and pressed between two clean glass plates. OCD was recorded when at least 90% of the grains no longer had opaque core of uncooked kernel centers. The rice was then allowed to simmer for another two minutes to ensure that all of the endosperm of all grains had been gelatinized. The OCD including the additional two minutes of simmer was determined to be a total of 24 min of cooking time so that is what was used for this study, at a water-to-rice ratio of 2:1. Although there are more modern methods of determining OCD [39], this method is still accepted and widely used for its efficiency. Each of the five treatments of raw rice samples were placed in transparent glass bowls to be presented to the participants. Five sets of two samples were presented to participants one after another in random order according to William’s Latin Square design. In each set of two samples, the control (5%) was labeled control with the test sample labeled with a three-digit code. The five tests were as follows: (1) control (5%)–test (5%), (2) control (5%)–test (10%), (3) control (5%)–test (20%), (4) control (5%)–test (30%), and (5) control (5%)–test (40%).

### 2.3. Procedure

In response to the COVID-19 pandemic, as participants arrived for the test, their temperatures were taken, they were instructed to wash their hands, and social distancing of six feet was always enforced between participants. Once in the sensory booth, the tests began with the uncooked rice portion of the experiment. Two samples, one labeled control and the other labeled with a three-digit code, were presented five separate times to each participant. They were asked to consider all aspects of the rice samples and indicate the size of the difference between the two samples on a 10-cm line scale with no difference on one end of the scale and extreme difference on the opposite end of the scale. This type of question has been shown to be effective in quantitative descriptive analysis to understand the degree at which a difference is perceived by individual panelist [40].

Following the five sets of uncooked samples, the cooked samples were presented each in a plastic cup with a white plastic spoon and a plain cracker and cup of water for palate cleansing. The cooked samples were randomly presented to the participants one after another with the only controllable difference being the level of brokens in the cooked rice. The same five levels used in the uncooked portion of the test (5%, 10%, 20%, 30%, and 40%) were also used in this cooked rice portion of the test.

For each rice treatment, a series of questions were asked of the participants before they were presented their next sample. First, the participants were instructed not to taste the rice sample before answering questions using a 9-point hedonic scale from dislike extremely to like extremely about their perception of the appearance of the rice sample. Then, they were instructed to sniff the rice sample and determine their liking/disliking using the same 9-point hedonic scale of the overall aroma of the rice sample. This was not an aromatic rice, so we expected to see no differences. Next, they were instructed to taste the sample and rate the rice flavor intensity, hardness (firmness), and stickiness using a 7-point JAR (Just About Right) scale. They also rated, using the 9-point hedonic scale, the overall flavor, the texture (mouthfeel), and overall impression. Finally, the participants were asked to rate their willingness to purchase from extremely unlikely to purchase to extremely likely to purchase on a 9-point hedonic scale.

### 2.4. Statistical Analysis

Data were collected using Compusense Cloud^®^ (Compusense Inc., Guelph, ON, Canada) software and analyzed using XLSTAT (Addinsoft, Long Island, NY, USA) and JMP^®^ Pro (version 16.0, SAS Institute Inc., Cary, NC, USA). To determine whether the five milled-rice samples varying only with the inclusion percentage of broken rice could differ in terms of the degree of difference from the control, a two-way analysis of variance (ANOVA), treating “test sample” and “panelist” as fixed and random effects, respectively, was conducted. If a significant difference was identified, post hoc comparisons between the test samples were performed using Tukey’s honestly significant difference (HSD) tests.

To determine whether the five cooked-rice samples prepared using milled rice varying in their inclusion percentage of broken rice could also differ with respect to hedonic impression, sensory perception, and willingness to purchase, a two-way ANOVA, treating “test sample” and “panelist” as fixed and random effects, respectively, was conducted, along with post hoc comparisons using Tukey’s HSD tests, where appropriate. Additionally, for the JAR scale data, a penalty analysis was used to identify how much each sensory attribute affected overall liking of rice samples varying in their inclusion percentage of broken rice. In this analysis, JAR indicated that the percentage of JAR scores was greater than 70% and no more than 20% of responses were on either the minus (−) or the plus (+) side of the scale [41]. A partial least square regression (PLSR) analysis was conducted to determine the impacts of individual attribute likings on participants’ willingness to purchase cooked rice samples as a function of inclusion percentage of broken rice. Centered and scaled values of the independent and dependent variables were used along with a Leave-One-Out cross-validation method to determine the lowest number of factors required to both minimize root-mean PRESS (RM-PRESS) value and maximize percentages of variations explained for the independent X-variable and the dependent Y-variable [42]. Variable influence on projection (VIP) values and standard coefficients were used to determine meaningful contributors to willingness to purchase cooked rice samples [19]. While independent variables with VIP values above 0.8 were important, those below 0.5 were considered not to be relevant [43,44]. Positive and negative standardized coefficients of independent variables reflected positive and negative correlations of these variables with willingness to purchase cooked rice samples.

## 3. Results

### 3.1. The Degree of Noticeable Difference between Milled Rice Samples Varying in the Percentage of Broken Rice

**Figure 1:**
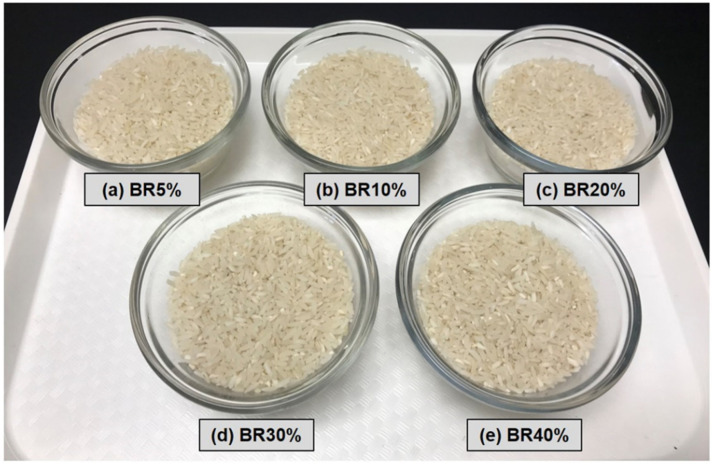
Display of rice with varying levels of brokens.

**Figure 2:**
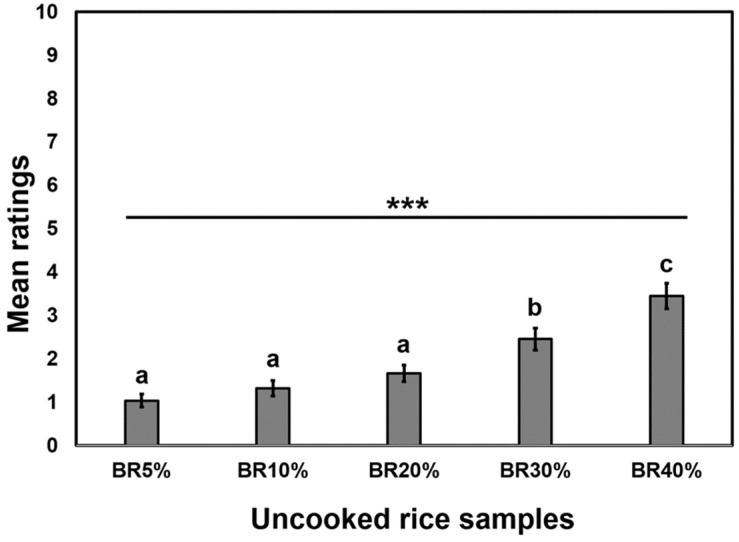
Mean comparisons with respect to the degree of difference between the control and five test samples ranging from 5%, 10%, 20%, 30%, and 40% for their inclusion percentage of broken rice (BR). *** represents a significant difference at *p* < 0.001. Means with the different letters represent a significant difference are significantly different (*p* < 0.05) using Tukey’s honestly significant difference (HSD) tests.

Participants initially evaluated the raw rice samples in the sensory laboratory (Figure 1). They rated the degree of difference (DOD) between the control and five test samples ranging from 5%, 10%, 20%, 30%, and 40% for their inclusion percentage of broken rice. Mean ratings of the DOD for individual test samples were below 3.5 on a scale ranging from 0 (no difference) to 10 (extreme difference), suggesting that these participants did not consider the broken rice-induced variations as reflecting pronounced differences when compared with the control (Figure 2). Even though the DOD ratings of the five test samples were relatively small (i.e., below 3.5), it should be noted that they were significantly different (*F* (4396) = 29.25, *p* < 0.001). More specifically, while participants were unable to differentiate rice samples that included broken milled rice ranging between 5% and 20% of their total weights (i.e., BR5%, BR10%, and BR20%), they were able to differentiate those samples from the control rice samples that included broken rice as either 30% (BR30%) or 40% (BR40%) of the total weight.

### 3.2. Broken Rice-Induced Variations in Hedonic Impression, Sensory Perception, and Willingness to Purchase with Respect to Cooked-Rice Samples

#### 3.2.1. Hedonic Impression

**Figure 3:**
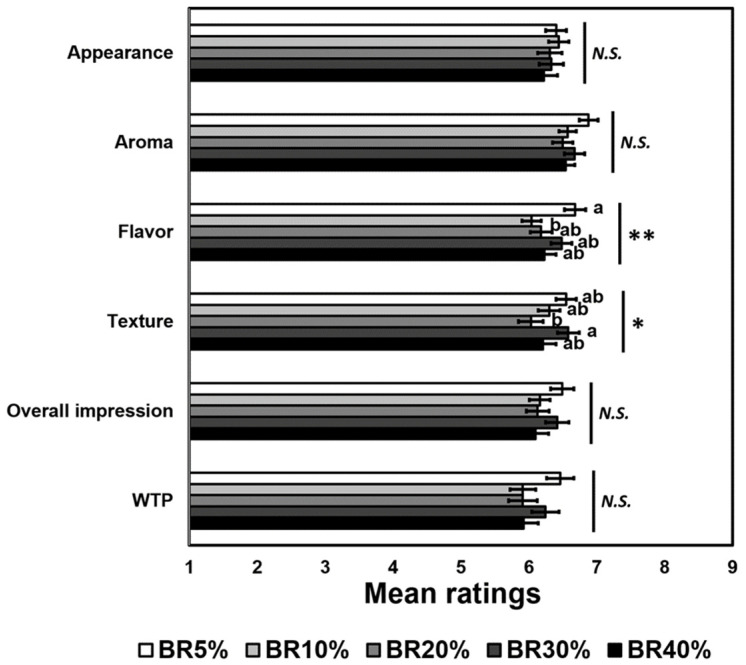
Mean comparisons with respect to hedonic impression and willingness to purchase (WTP) of the five cooked-rice samples with different levels of broken rice (BR) inclusion: 5%, 10%, 20%, 30%, and 40%. *N.S.* represents no significant difference at *p* < 0.05. * and ** represent a significant difference at *p* < 0.05 and *p* < 0.01, respectively. Means with the different letters for each attribute are significantly different (*p* < 0.05) using Tukey’s honestly significant difference (HSD) tests.

Figure 3 includes mean comparisons among the five cooked-rice samples with respect to hedonic impression expressed in terms of aroma, flavor, texture, and overall impression. The five cooked-rice samples differed significantly in flavor liking (*F* (4396) = 3.63, *p* = 0.006) and texture liking (*F* (4396) = 2.83, *p* = 0.03). More specifically, participants preferred flavors of cooked rice prepared using BR5% more than those of cooked rice prepared using BR 10%, but not for any of the other sample percentages. The participants also liked textural characteristics of cooked rice prepared using BR30% more than those of cooked rice prepared using BR20%. However, the five cooked-rice samples did not differ with respect to appearance liking (*p* = 0.81), aroma liking (*p* = 0.07), and overall impression (*p* = 0.18). This was encouraging in support of our “no perceived difference hypothesis”.

#### 3.2.2. Sensory Perception: Just-About-Right

For all five cooked-rice samples, nearly all participants considered flavor intensities (ranging between 2.40 and 2.64) of the test samples to be weaker than their JAR level (4-point), thereby reducing overall liking of cooked rice samples (Table 1). The mean-drop values of flavor JAR ratings were especially pronounced in the cooked-rice sample prepared using BR5% (Table 1). Although the five cooked-rice samples were found to differ in their mean ratings of flavor JAR (*F* (4396) = 2.42, *p* = 0.048), post hoc tests revealed no significant sample difference (*p* > 0.05). More than half of participants considered that firmness and stickiness intensities, respectively, were close to their preferred JAR levels, with more than 20% of total participants considering the five cooked-rice samples too firm (except for BR30%) and too sticky, resulting in a decrease in overall liking of cooked rice (Table 1). The five cooked-rice samples did not differ in their mean ratings of JAR with respect to firmness (*p* = 0.88) and stickiness (*p* = 0.59).

#### 3.2.3. Willingness to Purchase

**Figure 4:**
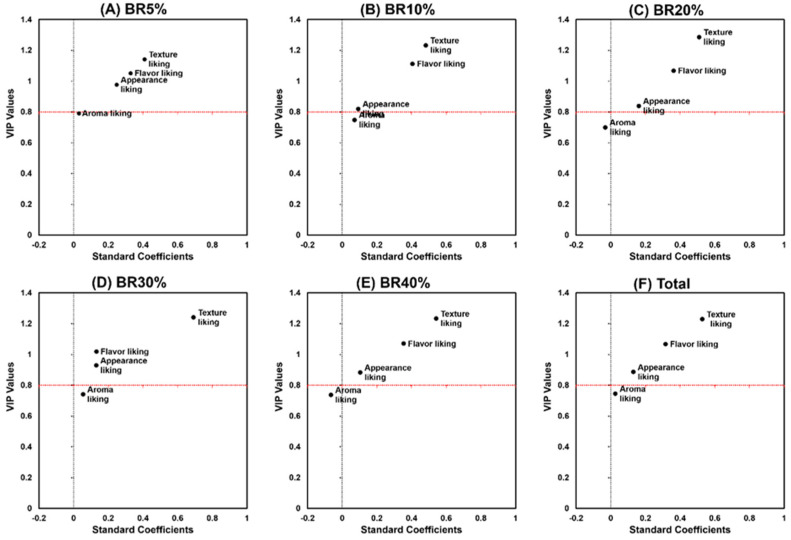
Variable influence on projection (VIP) and standard coefficient values of partial least squares regression (PLSR) with respect to willingness to purchase cooked rice samples prepared using raw rice varying in the inclusion percentage of broken rice (BR): 5%, 10%, 20%, 30%, and 40%. The horizontal dot line represents the cut-off VIP values of 0.8. Independent variables with VIP values above cut-off values of 0.8 were considered to be important. The positive or negative standard coefficient value of each attribute was considered to be positively or negatively associated with the willingness to purchase cooked rice samples, respectively.

The five cooked-rice samples did not differ in terms of willingness to purchasep (*p* = 0.07) (Figure 3). Partial least squares regression (PLSR) analyses revealed that three variables: appearance liking, flavor liking, and texture liking, played an important role in modulating these US consumers’ willingness to purchase cooked rice samples, regardless of the inclusion on increasing percentages of broken rice (Figure 4). Ara, 2003 conducted a WTP with routine rice consumers in two locations in the Philippines evaluating six rice quality attributes as to how these consumers perceived the benefits of purchasing organic rice. Ara found that how close consumers lived to the rice producing areas had a major impact on the value these consumers attributed to the reduction in pesticide usage on organically produced rice. Steur, et al. [45] conducted a WTP study of the value Chinese consumers perceived for rice fortified with folate known to diminish the risk of neural tube defects in the fetus. While neither of these studies addressed the quality impact of including increased percentages of broken rice, they both use WTP with un-trained consumers as a method of assessing routine rice consumers’ willingness to purchase rather than using trained sensory panels.

Interestingly, while both the VIP values and standard coefficients of “appearance liking” were found to be the largest in the cooked rice sample prepared using the lowest inclusion percentage of broken rice (BR5%), such impacts on consumer willingness to purchase were lower than those of “texture liking” and “flavor liking”. Overall, texture liking exhibited the largest VIP values and standard coefficients across the five cooked-rice samples, followed by flavor liking and appearance liking.

## 4. Discussion

Because this study was a consumer-focused study (i.e., the participants were rice-eating-consumers and not trained panelists), many of the points of analysis focused on willingness to purchase (WTP). Understanding why rice-eating consumers purchase the rice that they do was at the forefront of interest for the study, mainly how brokens affected rice consumers’ purchasing decisions. Testing respondents’ impressions of raw rice provided insight as to how the consumer viewed rice pre-purchase. This study was then able to compare those same respondents’ answers to questions about the cooked rice of the same levels of brokens to understand if the sentiments pre-purchase remained the same post-cooking.

Through observing consumers’ changing perceptions of uncooked rice with varying levels of brokens, an empirical trend showed that consumers can visually discern increased difference as percentage broken in the rice samples increased (Figure 1**).** The more brokens in the rice sample, the greater the difference consumer could discern between the test sample and the control. This trend was seen at every level of increased brokens, with significance observed (*p* value < 0.05) between 20% and 30% and between 30% and 40%. The test used a 10-cm line scale which has been shown to be effective at quantifying the difference between two samples while giving the participant the ability to make a judgement on a continuous, less limited scale [46]. Given that a “0” at the far-left end of the scale would mean “No Difference” and a “10” at the far-right end of the scale would mean “Extreme Difference”, the average scores were relatively low across all the treatments. The range of mean scores for the treatments was from 1.04 (5%) to 3.45 (40%), which shows that almost no differences were detectable in the 5% and only a moderately low amount of difference was detected in the 40%. Thus, even at 40% broken, there was not a huge amount of difference detected, which is understandable as all other controllable factors besides brokens were the same between the control and treatment samples.

The responses showing that consumers could tell the difference in increasing levels of brokens in rice led to the question of whether the brokens were a factor in their purchasing decision. Across many cultures and rice-eating-populations, people tended to view brokens as being of lower quality and prefer rice with lesser brokens [47]. In some cultures, brokens are perceived positively because of their unique cooking attributes or because of their association with certain (imported or domestic) rice which might be preferred [48]. There was also a large population of rice-eating-consumers who were simply unaware of broken rice in their typical rice purchasing decisions [49]. This test was conducted in Arkansas in the United States, where rice consumption per capita is relatively low. Because of the population tested, it was assumed that participants either had a negative connotation or no connotation concerning broken rice. Knowledgeable consumers could then use this information when prioritizing the level of brokens in their rice. This indicated that seeing increased levels of brokens in raw rice could have a negative impact on consumers’ WTP, as this trend was consistent across all samples.

This knowledge is coupled with the extensive questions throughout the rest of the study pointed to these participants’ general lack of awareness of varying levels of brokens in the samples. As seen in Figure 2, there was no linearity throughout the increasing levels of brokens, no matter which response was being evaluated. In every hedonic question, 5% was rated higher than 40%, but this difference was not significant throughout. Figure 4 demonstrates that there was no significance in any of the diagnostic questions, but it did show a trend that Firmness slightly decreased as percentage broken increased and Stickiness slightly increased as percentage broken increased. This led to an understanding that increased brokens create rice that kernels were more sticky and less individual kernels, which could be desirable or undesirable depending on preference and recipe. Overall, the hedonic responses demonstrated that there was a relatively small difference in perception between 5% and 40% broken compared to the eight-fold difference in brokens. This discrepancy in perception is not in line with the relative cost difference. Brokens are often sold at significant discounts (almost 42% in 2020) [7] because broken rice is typically used for products that are not meant for direct human consumption [50] despite them being equally as nutritious and not significantly different in sensory perception from head rice as shown in this study.

The size difference (raw) had a negative correlation with WTP of cooked rice (data not shown), with the inclusion of brokens in uncooked rice having a disproportionately negative impact on rice-eating-consumers’ WTP. Two additional WTP studies, one with rice consumers in the Philippines [35] and one with Chinese consumers [45], indicated support for the WTP method. To better understand this conclusion, consider in this scenario that brokens are sold from manufacturer to the retailer at a 40% discount and whole rice in this example is sold at USD 4.00/kg: Rice A contains 5% broken and sold at USD 3.92, whereas Rice B with 40% broken is sold at USD 3.36. That is a USD 0.56/kg increase or a 14% plus increase in price from Rice B to Rice A for the retailer. That retailer would have to sell the Rice A at a considerably higher price (at least 14% higher) to make the same profit as Rice B, which is a lot of money when selling rice in terms of thousands of kilograms. But, according to this study, it appeared that the average rice-eating consumer could only detect a small, sometimes negligible, difference between the two. If reasonable consumers would pay less for a product of perceived equal value, why would the supplier not want to sell the 40% broken rice at a higher price. This is likely only because of the perceived difference in the raw rice as seen in the first part of the study.

## 5. Conclusions

The conclusions from this study should not be overstated but will hopefully spark additional research into consumers’ perception of broken rice and an increased understanding of how to leverage opportunities for increased human consumption of a minimally used resource, broken rice. This study adds to a growing interest and understanding of how broken rice factors into all aspects of the supply chain from farm to rice miller, to retailer and finally to consumer. Broken rice is sold at significant discounts of 20% to 40% from rice suppliers, are cheaper to retailers and consumers because of their perceived “poor” appearance. The discounts are not because of the poor-quality attributes of cooked rice, as this study (among others) has shown. Many rice-eating-consumers have a difficult time discerning differences in rice of different percentage broken, especially if it is only a small percentage difference. Rice is almost always purchased in its raw form. Therefore, it is essential that consumers understand that despite their being able to perceive differences in the percentage of broken rice in the raw form and corresponding differences in price, there will probably be negligible difference in the cooked rice they serve their families. Improved understanding of why the poor appearance of brokens rice in its raw form plays such a profound role on price despite these US consumers’ impression of brokens in cooked rice as not having a strong impact on cooked rice quality. Trained panels could be used in future studies to increase specific knowledge on how the same sample of broken rice in its raw and cooked forms are perceived. Finally, further studies should seek to understand the breakpoint of acceptance (for both raw and cooked) for brokens with a narrower range of variation, as this study had a wide range of 5–40%. Additionally, research on changing the cooking parameters to find optimal preparation conditions that are varied according to the percentage brokens could be conducted.

In conclusion, additional research needs to be conducted to further our understanding of how consumers understand how the appearance of their raw rice impacts the cooked rice quality. This may eventually lead consumers to preferentially purchase discounted rice with increased percentage of broken rice. Supporting research in this vital area could eventually lead to increased human consumption of broken rice at discounted prices which would be vital for those facing hunger with limited means.

## Figures and Tables

**Table 1:** Summary of penalty analysis for the 7-point Just-About-Right (JAR) scale attributes in the 5 cooked rice samples evaluated by 100 rice consumers in the United States.

Attributes	Cooked Rice Samples ^1^					
	BR5%	BR10%	BR20%	BR30%	BR40%	Total
Flavor JAR	−97.0% (4.1) ^2^	−96.0% (1.2)	−96.0% (1.4)	−98.0% (2.0)	−92.0% (1.2)	−95.8% (1.7)
Firmness JAR	+21.0% (2.2)	+26.0% (1.1)	+25.0% (1.4)		+23.0% (1.5)	+22.8% (1.5)
Stickiness JAR	+38.0% (1.3)	+33.0% (0.9)	+31.0% (2.5)	+32.0% (1.3)	+34.0% (2.2)	+33.6% (1.6)

^1^ Cooked rice samples prepared using milled rice varying in the inclusion percentage of broken rice (BR): BR5%, BR10%, BR20%, BR30%, and BR40%; ^2^ Percentage (%) of participants (mean drop of overall liking): minus (−) and plus (+) symbols represent “too little” and “too much”, respectively.

## Data Availability

Data available from authors on reasonable request.
